# Clinical utility of the Pediatric Bowel Management Scoring Tool in guiding management of childhood constipation—a prospective, multicenter study

**DOI:** 10.1007/s00431-026-06901-x

**Published:** 2026-04-07

**Authors:** Michelle N. Bloem, Lexi D. E. Kassies, Shaista Safder, Udo Rolle, Giovanni Mosiello, David Marshall, Ilan J. N. Koppen, Marc A. Benninga

**Affiliations:** 1 Department of Pediatric Gastroenterology and Nutrition, Emma Children’s Hospital, Amsterdam UMC, University of Amsterdam, Amsterdam, The Netherlands; 2 Amsterdam Gastroenterology Endocrinology Metabolism Research Institute, Amsterdam UMC, Amsterdam, The Netherlands; 3 Amsterdam Reproduction and Development Research Institute, Amsterdam UMC, Amsterdam, The Netherlands; 4 Department of Pediatric Gastroenterology, Arnold Palmer Hospital for Children, Orlando Health, Orlando, FL USA; 5 Department of Pediatric Surgery and Paediatric Urology, Goethe-University Frankfurt, 60590 Frankfurt, Germany; 6 Department of Surgery, Division of Urology, Bambino Gesù Pediatric and Research Hospital, 00165 Rome, Italy; 7 Department of Pediatric Surgery and Pediatric Urology, Royal Belfast Hospital for Sick Children, Belfast, BT12 6BE UK

**Keywords:** PBMST, Transanal irrigation, Healthcare practitioner

## Abstract

To evaluate the clinical utility of the Pediatric Bowel Management Scoring Tool (PBMST) by assessing its association with healthcare practitioner (HCP) treatment decisions and patient-reported quality of life in children with constipation using transanal irrigation (TAI). A multicenter prospective cohort study was conducted in 121 children (4–18 years) with functional or organic constipation, either newly initiating (NTP) or currently receiving (CTP) TAI. The PBMST and PedsQL™ 4.0 quality of life questionnaires were completed at baseline and one-month follow-up. PBMST outcomes range from 0 to 22; higher scores indicate worse bowel management. HCPs, blinded to PBMST scores, classified treatment strategies as adequate, sub-optimal, or inadequate. Associations between PBMST scores and HCP treatment decisions were analyzed using multiple linear regression, adjusting for age, sex, and participant type (NTP or CTP). Correlations between PBMST and PedsQL™ scores, as well as associations between TAI adherence, duration of TAI use, and predictors of high adherence (MARS-5 ≥ 23), were examined. Survey completion rates were 93% at baseline and 88% at follow-up. Higher PBMST scores were associated with HCP-rated inadequate treatment (*B* = 2.44, SE = 0.72, *p* = 0.001), independent of age, sex, and participant type (NTP or CTP). PBMST scores were negatively correlated with PedsQL™ scores (*r* =  − 0.47, *p* < 0.001). No significant associations were found between adherence and TAI duration or PBMST scores. Lower adherence to TAI was associated with older age and participants reporting that TAI was too time-consuming.

*Conclusion*: The PBMST is a valid tool reflecting symptom severity, correlating with HCP treatment decisions and patient-reported quality of life in children with functional or organic constipation using TAI.

**Supplementary Information:**

The online version contains supplementary material available at 10.1007/s00431-026-06901-x.

**Table:** 

**What is Known:** • *The Pediatric Bowel Management Scoring Tool (PBMST) was developed to assess bowel management in children with constipation. However, its clinical utility in guiding treatment has not been fully established.*	
**What is New:** • *PBMST scores are significantly associated with healthcare practitioner treatment decisions in children using transanal irrigation.* • *PBMST scores strongly correlate with patient-reported quality of life, supporting its value in clinical assessment.*	

## Introduction

Constipation is common in childhood and can lead to pain, fecal incontinence, and reduced quality of life(1). In over 95% of cases, no underlying disease is found(2), and these children are diagnosed with functional constipation (FC) according to the Rome IV criteria(3, 4). With a worldwide pooled prevalence of 9.5% in children and adolescents, and due to its often chronic nature, constipation results in recurrent visits with health care practitioners (HCPs), leading to high health care utilization and costs.(5–8).

Conventional treatment consists of non-pharmacological interventions, such as education and toilet training, and pharmacological treatment with oral and/or rectal laxatives(9). In children who remain symptomatic despite treatment, transanal irrigation (TAI) can be applied as a step-up approach (Appendix)(10). TAI entails large-volume water irrigation of the rectum and colon via the anus to prevent accumulation of large quantities of stools(10).

**Fig. 1:**
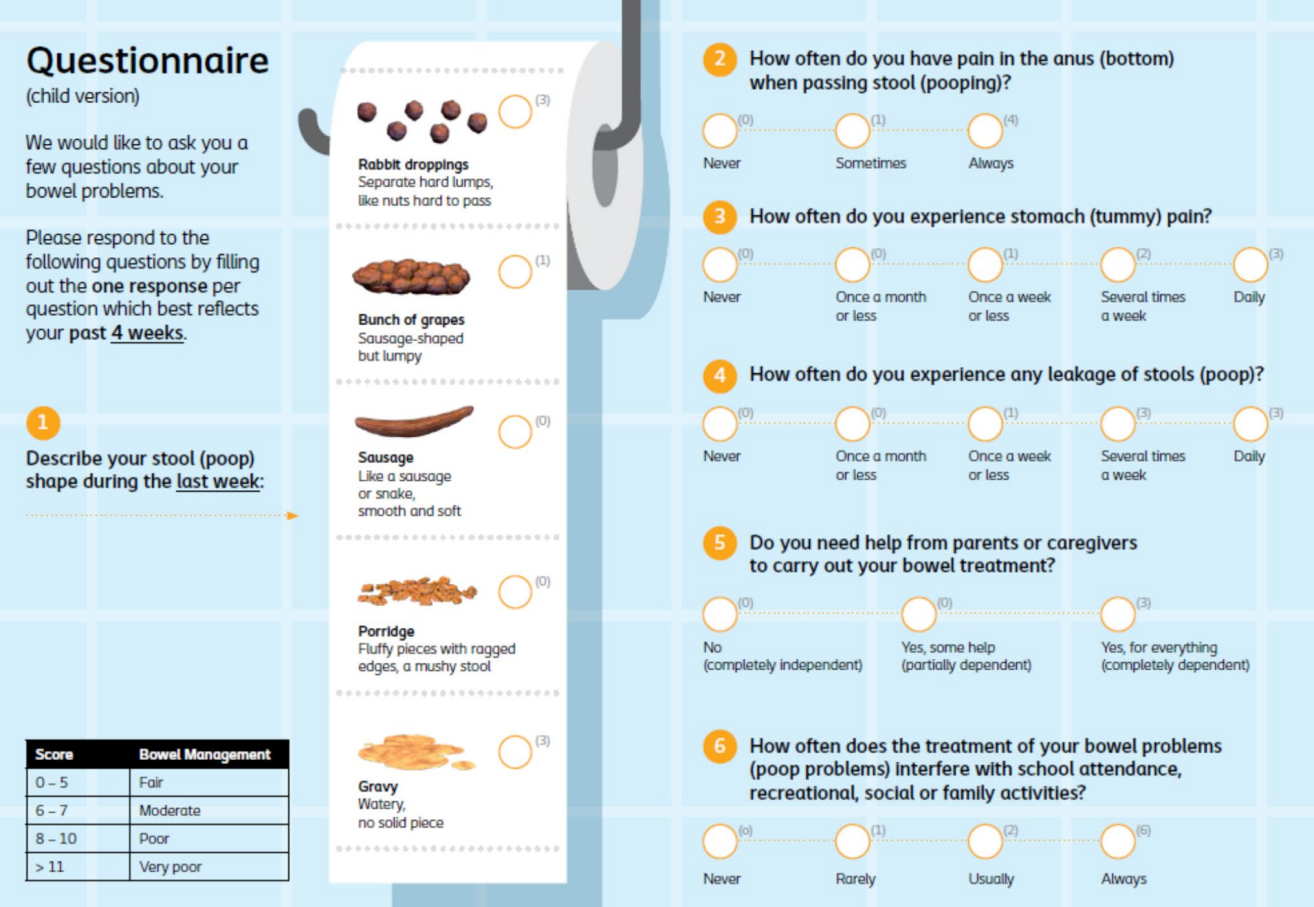
Pediatric Bowel Management Scoring Tool, child version (8–18 years old)(13). The PBMST is a six-item questionnaire directed at children (8–18 years old; self-reported) or their legal guardian (4–8 years old; proxy-reported), assessing stool shape, anorectal pain, abdominal pain, fecal incontinence frequency, caregiver assistance, and interference with social activities, yielding a score categorized as fair (0–5), moderate (6–7), poor (8–10), or very poor (≥ 11) bowel management

Currently, evaluation of the effectiveness of treatment strategies applied in children and the timing of progressing to a next step in treatment are determined based on medical history, physical examination, and expert opinion. In clinical practice, the use of a daily symptom diary is often useful in guiding the HCP to determine the bowel management strategy in children. In adults with chronic constipation, validated scoring tools have been developed to evaluate management of constipation(11, 12). Recently, a scoring system was created to help HCPs evaluate bowel management strategies in the pediatric population; the Pediatric Bowel Management Scoring Tool (PBMST; Fig. 1)(13). The PBMST is a short six-item questionnaire for children assessing symptom severity and impact on daily life. Although the PBMST has undergone validation in children diagnosed with constipation of functional or organic etiology(13), it is a newly devised instrument, and several aspects of its clinical utility and applicability have not yet been investigated. The original validation focused primarily on the tool’s psychometric properties and correlation with a single quality of life question, rather than a validated health-related quality of life instrument. The PBMST’s performance in clinical decision-making, responsiveness to change over time, and integration into routine care have not yet been evaluated. Therefore, the aim of this study was to assess the clinical utility of the PBMST in routine clinical practice for children with constipation by examining the association between PBMST scores, HCP’s treatment decisions, and patient-reported quality of life in children with either functional or organic constipation managed with TAI.

## Materials and methods

### Study design and participants

This multicenter, international, prospective observational survey study evaluated the clinical utility of the PBMST in children (4–18 years old) with either functional (fulfilling the Rome IV criteria for constipation) or an organic cause of constipation (e.g., anorectal malformations, neurogenic bowel, Hirschsprung disease) treated with TAI(13). Participants were recruited between May 2024 and March 2025 from Emma Children’s Hospital (Amsterdam, The Netherlands) and Orlando Health Arnold Palmer Hospital for Children (Orlando, USA).

Patients were eligible if they were initiating or already receiving TAI. Concomitant use of laxatives or other agents affecting gastrointestinal motility was allowed and recorded. Exclusion criteria were unwillingness to initiate TAI, lack of access to a personal email address, or insufficient Dutch or English proficiency. Children who discontinued TAI after enrollment were not excluded. Participants were classified as new TAI patients (NTP; initiating TAI) or current TAI patients (CTP; receiving TAI for at least three months, minimum three times per week at baseline). At baseline (*t* = 0), eligible patients scheduled for a clinical or telephone appointment were invited to participate by phone or in person. Follow-up was conducted by phone or in person, according to standard clinical practice after 1 month. Due to the observational nature of this study and the lack of previous literature, no formal statistical calculation was performed to determine the sample size. In consultation with a statistician, we estimated that enrolling 100 patients would be sufficient to ensure representation across all PBMST categories and HCP decision-making groups, providing a robust base for logistic regression analysis.

## Ethics approval

This study was conducted in accordance with institutional guidelines and the Declaration of Helsinki, was approved as non-WMO by the Amsterdam UMC Medical Ethics Committee (METC; reference: 2023.0794), and involved no risks beyond standard clinical care. All participants provided informed consent.

### Outcomes

The primary outcome was the association between PBMST total score (range 0–22) and HCP bowel management treatment decision at baseline (*t* = 0) and 1-month follow-up (*t* = 1), with both HCPs and patients blinded to PBMST results. Secondary outcomes included (1) association between PBMST categories (0–5: fair, 6–7: moderate, 8–10: poor, ≥ 11: very poor) and HCP treatment strategy at *t* = 0 and *t* = 1; (2) correlation between PBMST total score and participant quality of life (PedsQL™ Generic Core Scales 4.0, 23 questions, range 0–100)(14) at *t* = 0 and *t* = 1; (3) association between self-reported TAI adherence (MARS-5, range 5–25) and time on TAI, measured at *t* = 0 for CTPs and *t* = 1 for NTPs; (4) association between MARS-5 adherence and PBMST total score at *t* = 0 for CTPs and *t* = 1 for NTPs; and (5) identification of predictors of high TAI adherence (MARS-5 ≥ 23 consistent with the threshold used in previous literature(15)), including PedsQL™ score, PBMST score, age, sex, type of bowel dysfunction, and patient-reported adherence barriers.

### Data management

Data were prospectively collected by treating physicians and participants using validated and study-specific questionnaires and entered into Castor Electronic Data Capture. Collected data included patient characteristics, PBMST scores, MARS-5 adherence, HCP assessments, a self-developed TAI questionnaire, and PedsQL™ Generic Core Scales 4.0(14). Data were pseudonymized and securely stored per institutional and GDPR guidelines. Data corrections included reclassification of treatment decisions and adherence barriers to ensure consistency with operational definitions.

### Statistical analysis

Analyses were performed using RStudio 4.3.2 with *α* = 0.05. Descriptive statistics and histograms were used for exploratory analysis. Multiple linear regression assessed the association between PBMST total score (0–22) and HCP treatment decision (adequate vs. inadequate), adjusting for age, sex, and participant type. Regression assumptions were checked via diagnostic plots. For secondary outcomes, contingency tables and bar charts were used for categorical associations, and Pearson’s correlation coefficients for continuous variables. TAI usage characteristics were assessed descriptively at *t* = 1; if *t* = 1 data were missing, baseline (*t* = 0) data were used. Association between MARS-5 adherence and TAI duration was assessed using Pearson’s *r*, stratified by group (NTP/CTP) and time point. Predictors of high adherence (MARS-5 ≥ 23) were identified using multivariable logistic regression, including PedsQL™, PBMST, age, sex, bowel dysfunction type, and patient-reported barriers. A complete-case approach was used, including only participants with available data for all variables in the model. Continuous variables (PBMST, PedsQL™, age) were assessed for normality, and categorical variables were evaluated for balance between groups. Multicollinearity was checked using variance inflation factors (VIFs). Model selection used backward stepwise AIC, with model performance assessed by AUC and Hosmer–Lemeshow test; results are reported as odds ratios with 95% CIs. Continuous variables were assessed for normality; categorical variables were summarized by frequency. Group comparisons used Fisher’s exact, chi-square, or Mann–Whitney *U* tests as appropriate. No adjustment for multiple comparisons was made; secondary and exploratory analyses are considered hypothesis-generating. Wilcoxon signed-rank test assessed change in PBMST over time.

## Results

### Population characteristics

**Fig. 2:**
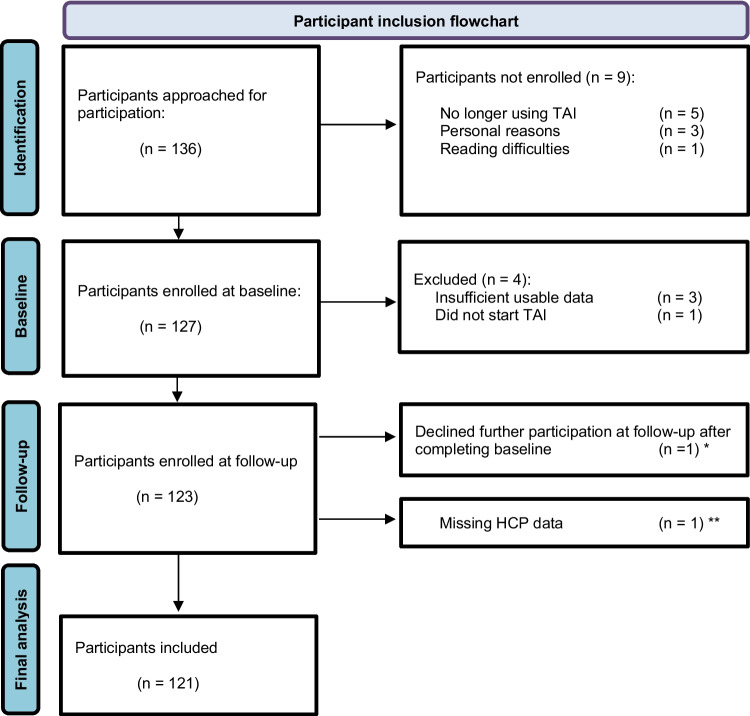
Participant inclusion flowchart. * Recorded as CTP and retained for baseline (*t* = 0) analysis only. ** Excluded for primary outcome 1 and secondary outcome 1 analyses, because the healthcare provider did not complete the decision-making questionnaire, making it impossible to compare PBMST and HCP decision data

Of 136 eligible children, a total of 127 children were enrolled, and 121 children were included in the final analysis (Fig. 2). Nine (7.4%) were aged 4 years, 26 (21.5%) aged 5–7 years, and 86 (71.1%) aged 8–18 years. In the cohort, 83 were CTP (69%) and 38 NTP (31%). No significant differences were observed between CTP and NTP groups regarding age category, sex, age at consultation, weight, or type of bowel dysfunction (Table 1). Children were recruited mainly in the Netherlands (*n* = 113, 82 CTP, 31 NTP) and the USA (*n* = 8, 1 CTP, 7 NTP). An organic cause of constipation was present in 5 (62.5%) of the 8 children recruited in the USA and in 14 (12.4%) of the 113 children recruited in the Dutch hospital. Among CTPs (*n* = 83), the median duration of TAI use at inclusion was 2.1 years [0.7–3.6]. At baseline, the majority (65.1%) irrigated ≥ 5 days a week, and 77.1% used oral laxatives as concomitant treatment. PBMST score categories differed significantly (*p* = 0.031), with a higher proportion of NTPs classified as having very poor scores (47.1%) compared to CTPs (24.7%).

**Table 1:** Patient characteristics per participant type at baseline

Characteristics	CTP (*N* = 83)	NTP (*N* = 38)	*p*-value
Age at consult—yrs	10.57 [7.67, 13.20]	10.23 [7.44, 14.06]	0.987
Age category—no. (%)	0.449		
4 years old 5–7 years old 8–18 years old	5 (6.0) 20 (24.1) 58 (69.9)	4 (10.5) 6 (15.8) 28 (73.7)	
Sex, male—no. (%)	46 (55.4)	20 (52.6)	0.929
Site—no. (%)			
USA, Orlando Health Netherlands, AUMC	1 (1.2) 82 (98.8)	7 (18.4) 31 (81.6)	
Bowel dysfunction—no. (%)			
Anorectal malformations Functional constipation Hirschsprung disease Neurogenic bowel disorders Other	1 (1.2) 72 (86.7) 4 (4.8) 4 (4.8) 2 (2.4)	0 (0.0) 30 (78.9) 1 (2.6) 4 (10.5) 3 (7.9)	
TAI system—no. (%)			
Does not irrigate (yet) Navina Peristeen Q-fora Other	0 (0.0) 17 (20.5) 44 (53.0) 18 (21.7) 4 (4.8)	38 (100.0)** - - -	
Time since start irrigation—yrs	2.10 [0.71, 3.62]	*-*	
TAI irrigation volume—ml/kg	15.71 [13.33, 20.00] *N* = 66	*-*	
TAI frequency—no. (%)			
Does not irrigate (yet) Every day 5/6 days per week Every 2 days Once a week Other	0 (0.0) 37 (44.6) 17 (20.5) 12 (14.5) 2 (2.4) 15 (18.1)	38 (100.0)** - - - - -	
TAI concomitant treatment – no (%)			
Does not irrigate (yet) Oral laxatives None Other	0 (0.0) 64 (77.1) 15 (18.1) 4 (4.8)	38 (100.0)* - - -	
PBMST category—no. (%)	0.031		
Fair (0–5) Moderate (6–7) Poor (8–10) Very poor (≥ 11)	31 (38.3) 11 (13.6) 19 (23.5) 20 (24.7)	5 (14.7) 3 (8.8) 10 (29.4) 16 (47.1)	

Data are reported as median [interquartile range].* CTP* current transanal irrigation patient, *NA* not available, *NTP* new transanal irrigation patient, *TAI* transanal irrigation, *PBMST* Pediatric Bowel Management Scoring Tool. *Weight at 1-month follow-up used. **This question has not been asked to NTP’s at baseline considering they are initiating treatment

### Association between PBMST and HCP treatment decision at baseline and follow-up

At baseline (*n* = 107), children whose treatment was rated as inadequate by HCPs had significantly higher PBMST *scores* than those with adequate treatment (*B* = 2.44, SE = 0.72, *p* = 0.001). Increasing age showed a trend toward lower PBMST scores with increasing age (*B* =  − 0.20, SE = 0.11, *p* = 0.058), while sex and participant type (CTP/NTP) were not significant predictors. At follow-up (*n* = 91), results were similar: HCP-rated inadequate treatment remained significantly associated with higher PBMST scores (*B* = 2.47, SE = 0.56, *p* < 0.001), and older age was associated with lower PBMST scores (*B* =  − 0.21, SE = 0.10, *p* = 0.029). Sex and participant type were not significantly associated with PBMST scores.

Higher PBMST *categories* were associated with a lower proportion of adequate treatment and a higher likelihood of treatment intensification by HCPs at both baseline and follow-up (Table 2). This trend confirms that increasing PBMST scores consistently aligned with HCP decisions to intensify or change therapy.

**Table 2:** Contingency table of PBMST score with HCP treatment decision at baseline (above) and follow-up (below)

		**Treatment decision**				**Total**
		Category 1: adequate treatment (weaning or no change)	Category 2: discussion but no change in treatment	Category 3: inadequate treatment (step-up or change)	Category 4: Other*	
PBMST score NTP/CTP	0–5 (Fair)	22	4	9	1	36
	6–7 (Moderate)	7	1	6	0	14
	8–10 (Poor)	10	1	17	0	28
	≥11 (Very poor)	5	3	26	0	34
Total		44	9	58	1	112

		**Treatment decision**				**Total**
		Category 1: adequate treatment (weaning or no change)	Category 2: discussion but no change in treatment	Category 3: inadequate treatment (step-up or change)	Category 4: Other**	
PBMST score NTP/CTP	0–5 (Fair)	36	4	7	1	48
	6–7 (Moderate)	15	2	6	1	24
	8–10 (Poor)	10	2	5	0	17
	≥11 (Very poor)	2	5	10	0	17
Total		63	13	28	2	106

*CTP* current transanal irrigation patient, *NTP* new transanal irrigation patient, *PBMST* Pediatric Bowel Management Scoring Tool

*Other, at t = 0, includes: (1) increase in loperamide dosage

**Other, at *t* = 1, includes: (1) initiation of home care due to family-related challenges; (2) temporary discontinuation of treatment during a gastroenteritis episode, followed by gradual resumption

### Association between PBMST scores and health-related quality of life

**Fig. 3:**
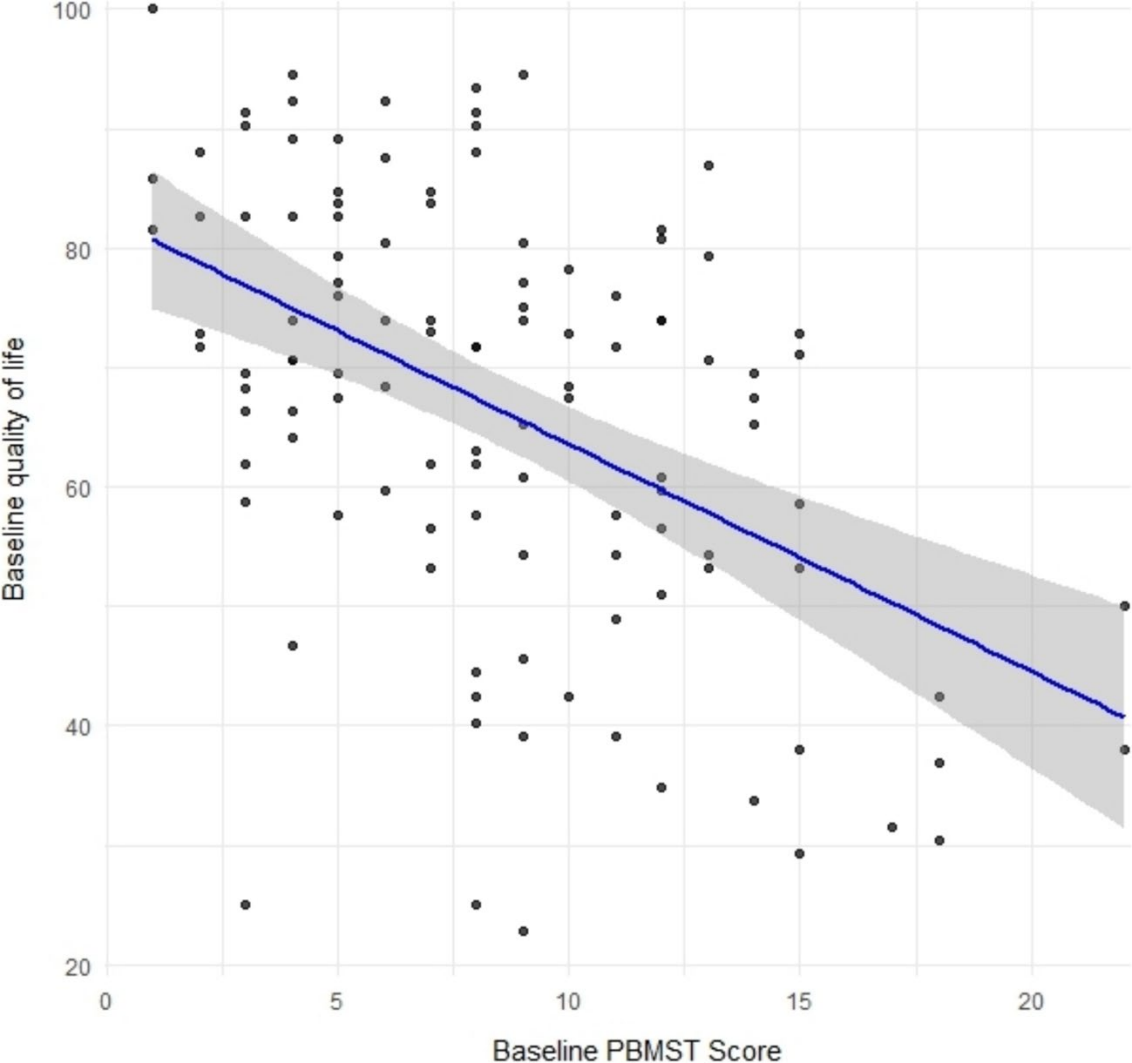
Correlation between PBMST Score and PedsQL total score at baseline. Each point represents an individual participant at baseline. The blue line represents the fitted linear regression line, with the shaded area indicating the 95% confidence interval. Higher PBMST scores, indicating more severe bowel management difficulties, were associated with lower-health related quality of life, as reflected in lower PedsQL™ scores

A moderate, statistically significant negative correlation between PBMST total scores and PedsQL™ 4.0 Generic Core Scales at both baseline (*n* = 113, *r* =  − 0.478, 95% CI [− 0.61, − 0.32], *p* < 0.001) and follow-up (*n* = 105, *r* =  − 0.473, 95% CI [− 0.61, − 0.31], *p* < 0.001) was observed, indicating that higher PBMST scores were associated with lower health-related quality of life (Fig. 3). Comparable negative correlations were observed for both psychosocial (baseline *r* =  − 0.473; follow-up *r* =  − 0.470) and physical (baseline *r* =  − 0.359; follow-up *r* =  − 0.415) PedsQL™ subscales (all *p* < 0.001), confirming a consistent association between greater bowel management difficulties and reduced quality of life.

### Change in PBMST scores over time

A statistically significant improvement in PBMST scores was observed between baseline and follow-up (*p* < 0.001). Of 107 participants, 62 (58%) showed improvement, 21 (16.6%) had no change, and 24 (22.4%) experienced deterioration in PBMST scores. Among NTPs, 3 (10.7%) worsened and 23 (82.1%) improved. Among CTPs, 21 (26.6%) worsened and 62 (78.5%) improved.

### Characteristics of TAI usage

Among 121 participants, including new TAI users, 68 (56.2%) used the Peristeen® system, 19 (15.7%) Navina™, 14 (11.6%) Qufora®, and 5 (4.1%) other systems (Appendix). Data on the TAI system were missing in 15 users (12.4%). The mean irrigation volume was 17.3 ml/kg (SD 6.3; *n* = 59). Most participants irrigated daily (39.7%) or 5–6 days per week (19.8%). Of the participants, 41.3% were completely dependent on their parents, 28.9% were completely independent, and 17.4% were partially dependent. Median duration of TAI use was 1.1 years (IQR 0.10–3.18) for all participants (CTP and NTP). For CTPs only (*n* = 83), median duration at time of inclusion was 2.1 years [IQR 0.71–3.62]. At follow-up, oral laxatives were used concomitantly by 55.4%, while 27.3% received no additional treatment. Tap water was used by 86.8% as the primary irrigation fluid. TAI was reported as always successful by 71.9% of participants. At follow-up, fecal incontinence between irrigations was most frequently reported as “never” (54.5%), representing an improvement compared to baseline PBMST assessments of fecal incontinence frequency (Table 3). Most participants (80.2%) reported no TAI-related problems; among those reporting issues (*n* = 9), pain, incomplete evacuation, technical problems, and behavioral challenges were described. One patient required weekly scheduled hospital support with TAI under anesthesia due to behavioral problems. Detailed descriptive statistics at follow-up are provided in the Appendix.

**Table 3:** Distribution of PBMST items at baseline

PBMST item	Overall *n* = 121	FC *n* = 102	OC *n* = 19
Stool shape			
1. Hard lumps 2. Sausage but lumpy 3. Sausage, smooth 4. Fluffy 5. Watery Missing data	10 (8.3%) 15 (12.4%) 27 (22.3%) 45 (37.2%) 18 (14.9%) 6 (5.0%)	10 (9.8%) 14 (13.7%) 21 (20.6%) 37 (36.3%) 14 (13.7%) 6 (5.9%)	- 1 (5.3%) 6 (31.6%) 8 (42.1%) 4 (21.1%) -
Anorectal pain			
Never Sometimes Always Missing data	47 (38.8%) 55 (45.5%) 13 (10.7%) 6 (5.0%)	38 (37.3%) 45 (44.1%) 13 (12.7%) 6 (5.9%)	9 (47.4%) 10 (52.6%) - -
Abdominal pain			
Never Once a month or less Once a week or less Several times a week Daily Missing data	14 (11.6%) 16 (13.2%) 27 (22.3%) 36 (29.8%) 22 (18.2%) 6 (5.0%)	10 (9.8%) 14 (13.7%) 19 (18.6%) 33 (32.4%) 20 (19.6%) 6 (5.9%)	4 (21.1%) 2 (10.5%) 8 (42.1%) 3 (15.8%) 2 (10.5%) -
Fecal incontinence			
Never Once a month or less Once a week or less Several times a week Daily Missing data	30 (24.8%) 12 (9.9%) 17 (14.0%) 37 (30.6%) 19 (15.7%) 6 (5.0%)	27 (26.5%) 10 (9.8%) 16 (15.7%) 28 (27.5%) 15 (14.7%) 6 (5.9%)	3 (15.8%) 2 (10.5%) 1 (5.3%) 9 (47.4%) 4 (21.1%) -
Help from caregivers			
No (completely independent) Yes (partially dependent) Yes (completely dependent) Missing data	14 (11.6%) 34 (28.1%) 67 (55.4%) 6 (5.0%)	13 (12.7%) 29 (28.4%) 54 (52.9%) 6 (5.9%)	1 (5.3%) 5 (26.3%) 13 (68.4%) -
Interference with daily activities			
Never Rarely Usually Always Missing data	30 (24.8%) 18 (14.9%) 51 (42.1%) 16 (13.2%) 6 (5.0%)	25 (24.5%) 17 (16.7%) 44 (43.1%) 10 (9.8%) 6 (5.9%)	5 (26.3%) 1 (5.3%) 7 (36.8%) 6 (31.6%) -

*PBMST* Pediatric Bowel Management Scoring Tool, *FC* functional constipation, *OC* organic cause of constipation (e.g., Hirschsprung disease, anorectal malformations, neurogenic bowel disorders)

### Association between duration of TAI use, self-reported adherence, and bowel management

Correlations between duration of TAI use and MARS-5 adherence scores were weakly negative and not statistically significant (CTP: *p* = 0.170, 95% CI [− 0.36, 0.07]; NTP: *p* = 0.572, 95% CI [− 0.49, 0.29]; combined: *r* =  − 0.04, *p* = 0.692, 95% CI [− 0.23, 0.15]). Among NTP (*n* = 27), a moderate, statistically significant negative correlation indicated that higher adherence was associated with better bowel management outcomes (*r* =  − 0.367, 95% CI [− 0.66, 0.02], *p* = 0.006). No significant association was observed for CTP (*n* = 80).

### Predictors of high adherence to TAI

Predictors of low adherence were older age (OR = 0.89, 95% CI: 0.79–0.99, *p* = 0.034) and reporting “*TAI takes too long*” (OR = 0.09, 95% CI: 0.010–0.735, *p* = 0.025, Appendix). Other factors, including pain, motivation, bowel dysfunction type, sex, PBMST, and PedsQL™ scores, were not significant. The final model (AIC = 132.39) demonstrated poor-to-fair discrimination (AUC = 0.676), and adequate calibration (Hosmer–Lemeshow *X*(2) = 4.75, *p* = 0.191).

### *Association between behavioral problems, adherence*,* and efficacy.*

Of 121 participants, 38 (31.4%) had behavioral problems (ADHD, ASS, or other), 73 (60.3%) had none, and 10 (8.3%) had missing data. At baseline, low adherence to TAI was similar in children with and without behavioral problems (44.8% vs. 45.1%; OR = 0.99, 95% CI [0.36–2.72], *p* > 0.99). At follow-up (*n* = 103), children with behavioral problems showed slightly lower rates of low adherence (36.1% vs. 43.3%; (*p* = 0.533), OR = 0.743, 95% CI [0.292–1.836]), but this was not significant. No differences in TAI efficacy were observed at baseline (40.5% vs. 39.4% effective; OR = 0.96, 95% CI [0.40–2.34], *p* > 0.99) or follow-up (51.4% vs. 63.8% effective; OR = 1.65, 95% CI [0.67–4.10], *p* = 0.29). At follow-up, children with behavioral problems were more likely to have ineffective treatment decisions (48.6% vs. 36.2%; OR = 1.654, 95% CI [0.670–4.104]), although this trend was not significant. These findings suggest no association between behavioral problems, adherence, and TAI efficacy.

## Discussion

This study provides an evaluation of the clinical utility of the PBMST in guiding management decisions for childhood constipation in patients using TAI. Consistent with recent publications, structured bowel management tools have been shown to significantly improve evaluation of pediatric functional constipation(16, 17). While such tools emphasize standardized care, the present findings indicate that symptom scoring tools such as the PBMST can support real-time assessment and guide individualized treatment decisions. These results also highlight the importance of incorporating patient-perceived burden into adherence assessment, aligning with the previously published patient-driven constipation action plan(18). Integration of tools such as the PBMST into broader, personalized management frameworks may enhance both clinical decision-making and patient engagement, bridging the gap between clinician judgment and patient experience to facilitate shared treatment goals(16, 18, 19).

HCP’s assessment of “inadequate treatment” was associated with higher PBMST scores, indicating that patients with poorer bowel management were more likely to require intensified treatment. The PBMST closely reflected clinical judgment, supporting its validity as a decision-support tool. A 2.4-point difference in PBMST scores between “Adequate” and “Inadequate” treatment groups represents a clinically meaningful distinction, demonstrating strong alignment between HCP assessments and patient-reported outcomes and reinforcing the scale’s construct validity(20). Categorical analysis further showed that higher PBMST scores were consistently associated with increased rates of treatment intensification, with over 75% of patients in the “very poor” category receiving such decisions at both time points. These results underscore the PBMST’s utility in identifying patients who may benefit from closer monitoring or management adjustments. Older participants tended to report lower PBMST scores, possibly reflecting age-related adaptation or improved physiological control(21). No significant effects were observed for sex or participant type, indicating that the PBMST performs similarly in both new and established TAI users.

The high proportion of patients classified as receiving “inadequate treatment” may be partly explained by the inclusion of NTPs at baseline, who are automatically categorized as needing treatment intensification upon starting TAI. This classification may overestimate the proportion of inadequate treatment, as it reflects protocol-driven intensification rather than true treatment failure.

Although the PBMST was originally constructed using a single, non-validated quality of life question to assess symptom impact(20), its relationship with validated HRQoL measures had not been established. The PBMST score was based on associations between symptom questions and a self-developed QoL item, grouping patients by “no or little impact” versus “some or major impact” on QoL, rather than using established instruments such as the PedsQL™. In this study, moderate negative correlations between PBMST and PedsQL™ scores support the construct validity of the PBMST, with higher scores consistently associated with lower health-related quality of life (HRQoL). This aligns with previous research demonstrating the psychosocial burden of chronic bowel conditions in children with FC compared to healthy peers (65.6 vs. 86.1, *p*< 0.01)(22–24). Children using TAI report HRQoL scores similar to other pediatric constipation populations(15), suggesting TAI does not further reduce perceived quality of life relative to other management strategies. Greater independence in toileting is associated with higher HRQoL(15), likely reflecting both age and functional status.

No significant correlation was found between duration of TAI use and self-reported adherence, indicating that length of TAI use does not predict adherence. This suggests that adherence challenges persist regardless of treatment duration and are not necessarily resolved with increased familiarity. Although no prior studies have directly examined this relationship in children using TAI, existing pediatric adherence literature indicates that factors such as age, perceived treatment burden, family dynamics, and caregiver involvement are more influential on adherence than duration of treatment(25).

No significant differences in PBMST or PedsQL™ scores were observed between high- and low-adherence groups, indicating that clinical symptom burden and perceived quality of life do not directly influence adherence in this population. These findings suggest that adherence in children may be more strongly affected by practical and motivational factors than by symptom severity or well-being.

Older age and the perception that TAI takes too long were independent predictors of low adherence, while perceived ineffectiveness of TAI was not, likely due to sample size or overlap with other predictors. Reporting that TAI takes too long was associated with a 91% reduction in the odds of high adherence, highlighting the impact of treatment burden, and each additional year of age decreased adherence odds by 11.5%, possibly reflecting developmental changes and reduced parental oversight(26). The model’s discriminative ability was modest (AUC = 0.676), suggesting that additional factors such as mental health or family dynamics may influence adherence(27).

In this cohort, 71.9% of participants reported that TAI was always successful, a rate consistent with previous studies in pediatric FC populations, which have documented success rates between 60 and 80%(15, 20, 28, 29). Consistent TAI success is expected to facilitate improved stool evacuation and reduce episodes of fecal incontinence, potentially contributing to better quality of life, social participation, and emotional well-being. A small number of participants in our study reported experiencing pain or strong aversion to TAI. Others occasionally reported incomplete evacuation, nausea, or technical difficulties such as water leakage or balloon malfunction. These findings are consistent with previous reports showing that, although TAI is generally safe and effective for pediatric bowel dysfunction, pain and discomfort are relatively common (15, 28, 30, 31) and a small percentage of children may not tolerate the procedure or experience leakage of irrigation fluid (15, 28, 32). Although concerns about electrolyte imbalance due to water intoxication or addition of laxatives in the irrigation fluid have been raised(10, 33), this was not observed in our cohort. Serious complications are rare (10, 28, 34).

### Strengths and limitations

This is the first prospective study to evaluate the clinical utility of the PBMST in guiding treatment decisions for children using TAI. The multicenter, international design and inclusion of both current and new TAI patients enhance generalizability and applicability. Blinded decision-making minimized observer bias.

However, recruitment was predominantly from the Netherlands (> 90%), with limited inclusion from the USA and no enrollment from other planned centers due to delayed ethics approvals, reducing generalizability. The Dutch cohort mainly included patients with FC, due to having a specialized outpatient clinic primarily for FC, reflecting differences in clinic populations.

Another key limitation is the disproportionate scoring weight of the PBMST’s final item, "*How often does current bowel treatment interfere with school attendance, recreational, social or family activities?*”), where the most severe response (“*Always*”) contributes 6 out of the 22 total points. This may overemphasize perceived treatment burden and inflate total scores, particularly when families interpret “*Always*” as reflecting treatment frequency rather than distress. This may explain why some patients with high PBMST scores were still considered adequately treated by their HCPs. Conceptual overlap between PBMST and PedsQL™, especially regarding psychosocial impact, may have inflated correlations.

PBMST applicability is limited in non-toilet-trained children (i.e., with neurogenic bowel dysfunction, and in children with functional non-retentive fecal incontinence, as several items are difficult to answer reliably in non–toilet-trained children, increasing the risk of measurement bias.

The MARS-5 may underestimate adherence, as deviations from prescribed TAI regimens can reflect appropriate clinical adjustments rather than true nonadherence(15).

## Conclusion

This analysis demonstrates the clinical utility of the PBMST in guiding management of childhood constipation with TAI, with scores aligning with HCP decisions and negatively correlating with HRQoL. A follow-up study will focus on user experience, incorporating qualitative feedback from children, parents, and providers to inform tool refinement and implementation. Integrating standardized tools like the PBMST into routine care, alongside structured management protocols and patient-driven action plans, may enhance individualized care, support timely intervention, and reduce unnecessary healthcare visits. Rising hospitalization rates for fecal impaction in children underscore the need for preventive strategies.^35^ While adherence was not associated with symptom severity or treatment duration, it was negatively influenced by older age and perceived time burden, underscoring the need to address practical barriers. Overall, the PBMST shows promise as a decision-support tool and indicator of treatment response, warranting continued evaluation and broader implementation.

## Supplementary Information

Below is the link to the electronic supplementary material.

ESM 1 (DOCX 42.9 KB)

## Data Availability

All our data and meta data will be made available upon request.

## Abbreviations

AMC: Academic Medical Center
CTP: Current TAI patients, patients currently treated with TAI
FC: Functional constipation
HCP: Healthcare practitioner
IQR: Interquartile range
MARS: Medication Adherence Report Scale
NTP: New TAI patients, patients initiating treatment with TAI
OR: Odds ratio
PBMST: Pediatric Bowel Management Scoring Tool
SD: Standard deviation
SE: Standard error
TAI: Transanal irrigation
