# Supplementary material for: Clinical utility of the Pediatric Bowel Management Scoring Tool in guiding management of childhood constipation—a prospective, multicenter study
Source: Eur J Pediatr. 2026 Apr 7;185(5):241. doi: 10.1007/s00431-026-06901-x (PMC13056778; doi:10.1007/s00431-026-06901-x)
Supplement: Supplementary file 1 — (DOCX 42.9 KB) [file 431_2026_6901_MOESM1_ESM.docx]

**Appendix**

**Appendix 1.**

**Figure 1:** Bowel management pyramid for children with bowel dysfunction. *The pyramid illustrates treatment options for constipation, arranged by increasing invasiveness. The first level represents conservative medical approaches. ^a^Most novel pharmacologic therapies need further research, but may include prosecretory and serotonergic agents. TAI is considered as an additional conservative treatment intervention. A following step can consist of antegrade colonic enema or neuromodulation (abdominal transcutaneous electrical stimulation, percutaneous tibial nerve stimulation, or sacral neuromodulation [SNM]). ^b^SNM is not licensed for use in children in some regions. The final level in the pyramid involves resection or ostomy.* (*Adapted by Bloem et al. 2025^14^ from the Consensus Review of Best Practice of Transanal Irrigation in Children, Mosiello et al. 2017*^10^)

Appendix 2**. Logistic regression model assessing predictors of high adherence to TAI at t=1**

| **High adherence to TAI** | **Estimate** | **OR (95% CI)** | **p-value** |
| --- | --- | --- | --- |
| **Full model** | | | |
| Age | -0.085 | 0.919 [0.806; 1.047] | 0.202 |
| Sex, male | 0.199 | 1.220 [0.488; 3.050] | 0.671 |
| Bowel dysfunction type (ref: FC) |  | | |
| Hirschsprung disease  Neurogenic bowel disorders  Other | -0.372 0.820  -0.279 | 0.690 [0.062; 7.703]  2.271 [0.240; 21.456]  0.757 [0.078; 7.308] | 0.763  0.474  0.810 |
| PBMST score | 0.048 | 1.049 [0.913; 1.206] | 0.498 |
| PedsQL^TM^ score | 0.006 | 1.006 [0.978; 1.035] | 0.674 |
| Barrier: irrigation is painful | -0.452 | 0.637 [0.174; 2.328] | 0.495 |
| Barrier: takes too long / logistical barrier | -2.132 | 0.119 [0.013; 1.091] | 0.060 |
| Barrier: low motivation | -1.186 | 0.306 [0.043; 2.166] | 0.235 |
| Barrier: feels not effective | -1.051 | 0.350 [0.025; 4.926] | 0.436 |
| **Reduced model** | | | |
| Age | -0.122 | 0.885 [0.790; 0.991] | 0.034* |
| Barrier: takes too long / logistical barrier | -2.450 | 0.086 [0.010; 0.735] | 0.025* |

Abbreviations: FC = functional constipation, MARS = Medication Adherence Report Scale, OR = odds ratio, PBMST = Paediatric Bowel Management Scoring Tool, TAI = transanal irrigation. Of 121 participants, 105 were included in the analysis (42 low adherence, 63 high adherence).

Appendix 3**: TAI characteristics at follow-up**

| TAI characteristics | Overall n = 121 |
| --- | --- |
| TAI system – no. (%) |  |
| Peristeen  Navina  Q-fora  Other NA | 68 (56.2)  19 (15.7)  14 (11.6)  5 (4.1)  15 (12.4) |
| TAI irrigation volume – ml/kg (mean (SD)) | 17.33 (6.29) n=59 |
| TAI frequency – no. (%) |  |
| Every day  5/6 days per week  Every 2 days  Every 3 days  Once a week  Other  NA | 48 (39.7)  24 (19.8)  12 (9.9)  1 (0.8)  1 (0.8)  20 (16.5)  15 (12.4) |
| TAI assistance needed – no. (%) |  |
| Completely dependent  Completely independent  Partially dependent  NA | 50 (41.3)  35 (28.9)  21 (17.4)  15 (12.4) |
| Time since start irrigation – yrs. (median [IQR]) | 1.10 [0.10, 3.18] |
| TAI concomitant treatment – no (%) |  |
| Oral laxatives  None  Other  NA | 67 (55.4)  33 (27.3)  6 (5.0 15 (12.4) |
| TAI content used – no. (%) |  |
| Tap water  Saline  Laxative enema (Klyx)  Fluimucil  Bisacodyl/PEG/Glycerine  NA | 105 (86.8)  1 (0.8)  2 (1.7)*  2 (1.7)*  0 (0.0)  15 (12.4) |
| TAI success rate – no (%) |  |
| Always  Usually  Sometimes  Never  NA | 87 (71.9)  13 (10.7)  5 (4.1)  1 (0.8)  15 (12.4) |
| Spontaneous defecation between irrigations – no. (%) |  |
| Always  Usually  Sometimes  Never  NA | 12 (9.9)  13 (10.7)  33 (27.3)  48 (39.7)  15 (12.4) |
| Fecal incontinence between irrigations – no. (%) |  |
| Always  Usually  Sometimes  Never  NA | 11 (9.1)  13 (10.7)  16 (13.2)  66 (54.5)  15 (12.4) |
| Patient-Reported Problems – no. (%) |  |
| Always  Usually  Sometimes  Never  NA | 2 (1.7)  1 (0.8)  6 (5.0)  97 (80.2)  15 (12.4) |

Abbreviations: IQR = interquartile range, NA = number of missing observations (15, 12.4% across all variables), SD = standard deviation, TAI = transanal irrigation.

*In all 4 cases, the content was used alongside tap water everyday: one added Klyx daily, one added Klyx twice-weekly, another added Fluimucil once-weekly, and the last one added Fluimucil daily. This clarification ensures that these participants are correctly counted toward the total sample of 121.
